# Supplementary material for: Management of biliary stricture in patients with IgG4-related sclerosing cholangitis
Source: PLoS One. 2020 Apr 30;15(4):e0232089. doi: 10.1371/journal.pone.0232089 (PMC7192452; doi:10.1371/journal.pone.0232089)
Supplement: Attachment — Submitted filename: Response to Reviewers.docx [file pone.0232089.s001.docx]

Response to Reviewers

Reviewer #1:

1. The abstract was written not clearly enough, sometimes a little confusing. I don’t understand it until I read through the paper, especially the following descriptions in the results. The manuscript would be perfect if the abstract is modified properly.

- I modified the descriptions in the results to make the abstract easier to understand.

2. The authorship is confusing. Did not see the “*” after author’s names. All “¶” contributed equally? First 5 authors and last 6 authors?

- I rewrote the title page as shown in the policy. Symbol * means corresponding author and was marked in author’s name. Roles of authors with symbol ¶ were investigation and supervision. A Role of authors with symbol & was investigation.

Reviewer #2:

1. My concern is that sample size is too small, only three patients who had undergone EBD, developed bile-duct stones which is hard to draw rigorous conclusions.

- As you say, I think the sample size is too small for three patients who developed bile-duct stones. The result may vary if patients of ISC increase and are observed for longer follow-up period. I add the above statement to the discussion part.

2. Logistic regression can predict the risk of developing a given disease, which is based on observed characteristics of the patient. Therefore, the authors should use Logistic regression to predict the risk for bile duct stones.

- The results of univariate analysis to determine the risk factors for bile-duct stones showed that EBD administration, sustained biliary stenting and duration of EBD were risk factors. Other clinical parameters were not risk factors of bile-duct stones. Logistic regression analysis was attempted, but the number of patients who developed bile-duct stones was too small to be analyzed. The result may vary if patients of ISC increase.
